# Small-Diameter Tube Wall Damage-Detection Method Based on TE01 Mode Microwave

**DOI:** 10.3390/s22176476

**Published:** 2022-08-28

**Authors:** Meng Shi, Lijian Yang, Songwei Gao, Guoqing Wang

**Affiliations:** School of Information Science and Engineering, Shenyang University of Technology, Shenyang 110870, China

**Keywords:** small diameter pipeline detection, field distribution equation, communication mode, TE01 mode, return loss, reflection coefficient, frequency shift

## Abstract

Accidents occur frequently in urban gas pipelines, and pipeline damage detection is an important means of ensuring pipeline safety. Aiming at the problem that the small diameter pipeline is difficult to detect, this paper proposes a detection method for the inner wall damage of a small-diameter pipeline based on the TE01 mode microwave and uses the TE01 mode to detect the inner wall damage of the pipeline by the terminal short-circuit reflection method. By analyzing the transition of microwave propagation mode at the defect, based on the Maxwell equation and the field distribution equation of the TE01 mode microwave in the pipe and the pipe wall current equation, the microwave reflection coefficient at the defect is established when the microwave distortion modes at the defect are TE and TM modes. A small-diameter pipeline simulation model is established, and the influence of the electric field, magnetic field, wall current distribution, and reflected wave reflection coefficient in the pipeline when inner wall defects of different widths are analyzed using the finite integral theory during microwave detection of the TE01 mode. An experimental platform for the microwave detection of small-diameter pipes was built to detect defects on the inner walls of pipes with different widths. The results show that the inner wall defect causes the electric field, magnetic field, current propagation period, and energy distribution of the TE01 mode microwave propagated in the pipe to be distorted, and the microwave reflection coefficient and return loss exhibit a significant frequency shift with the change in the defect width. The experimental and simulation results had a good consistency.

## 1. Introduction

Currently, accidents occur frequently in urban gas pipelines, and damage to the inner wall of the pipeline is a key issue affecting pipeline safety. Pipeline inspection is an effective means of evaluating pipeline damage and safety performance. Online inspection of pipelines can repair hidden pipeline safety hazards and improve pipeline operational safety. It can prevent pipeline explosion accidents and effectively guarantee national energy security.

The city gas pipeline network diameter is small, the radius of curvature of the pipeline is small, and it is difficult to achieve a comprehensive detection of existing conventional external the inspection methods. A small pipe diameter, small radius of curvature, high requirements for internal detection technology, and internal detection equipment are prone to jamming. Small-diameter pipeline microwave-detection equipment is highly applicable and can be placed at one end of the pipeline microwave-detection probe to achieve small-diameter pipeline detection to solve the following problems: the small diameter of the pipeline equipment makes integration and the pipeline bending angle is too large, and the equipment is hard to move through. For small-diameter pipeline damage, online detection has a very good application prospects. Scholars have carried out extensive research on the propagation mechanism and detection methods of microwaves in materials. The microwave resonance detection method establishes a microwave resonance equation. With an increase in the defect depth, the resonance frequency decreases [1,2,3,4,5], and the defect location is evaluated by measuring the group velocity of microwave propagation in the time domain [6,7], multiple regression models [8,9,10], and microwave mode conversion [11,12,13]. Microwave inspection data are used to predict defect features [14,15,16,17] through microwave imaging [18,19], neural networks [20], and other methods. In terms of the microwave detection mechanism of damage to the inner wall of small-diameter metal pipelines, there is still a lack of complete theoretical support, and much research work is still needed on the propagation law of TE01 modal microwaves in the pipeline and the microwave detection mechanism for damage to the inner wall of the pipeline.

This paper aims at the problem of internal detection of small diameter urban gas pipelines; the urban gas pipeline network has problems such as small pipe diameter and small radius of curvature of the pipeline, and existing conventional external detection methods cannot easily achieve comprehensive detection. The microwave-detection equipment for small-diameter pipelines has strong applicability. A microwave-detection probe can be placed at one end of the pipeline to realize the detection of small-diameter pipelines, which solves the problems of difficult integration of small-diameter equipment and poor passing ability of pipelines with large bending angles. It has a very good application prospect. In this paper, according to the actual diameter of urban gas pipelines, a microwave internal detection model for small-diameter pipelines with a pipe diameter of 66 mm and a wall thickness of 7 mm was designed. The distribution of characteristic quantities of TE01 mode microwaves in the pipeline, such as electric field, magnetic field, and wall current, as well as the distribution of microwave modes at defects as well as the changes in reflection coefficient and return loss of microwave reflected waves. It provides a theoretical basis for the on-line detection of small-diameter pipelines in TE01 model city gas pipeline network.

## 2. Microwave Detection Mechanism of TE01 Mode in Small-Diameter Pipeline

**Figure 1:**
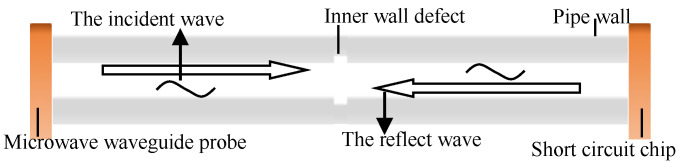
Schematic diagram of microwave inspection of pipeline inner wall damage.

As an electromagnetic wave, the microwave propagates radially along the pipeline. The microwave passes through the waveguide coaxial converter to convert the transverse electromagnetic wave (TEM wave) into the electromagnetic wave (TEmn wave) propagating in the pipeline. When TEmn is transmitted in the pipeline, the electric field has only a transverse component, and the electric field propagating along the axial direction of the pipeline is 0; a schematic diagram of the damage of the inner wall to the pipeline is shown in Figure 1.

As shown in Figure 1, the single-excitation short-circuit reflection method was used to excite microwaves at one end of the pipe through a waveguide probe, and a short-circuit piece was applied at the other end of the pipe. When the microwave propagates to the short-circuit piece, total reflection occurs; when there is a defect in the pipe, part of the energy is lost at the defect, and by analyzing the return loss, defect detection on the inner wall of the pipeline can be realized.

### 2.1. Propagation Mechanism of Microwaves in TEmn Mode in Pipes

**Figure 2:**
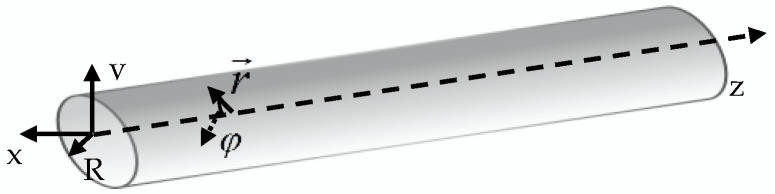
Pipeline coordinate system.

In the pipeline, the microwave propagates along the axial direction of the pipeline in TEmn mode. According to Maxwell’s equations, the microwave field distribution equation of the TEmn mode in a pipeline in a cylindrical coordinate system can be obtained. The pipeline coordinate system is shown in Figure 2.

The pipeline coordinate system is shown in the Figure 2; the inner diameter of the pipeline is R, the z-axis is the axial direction of the pipeline, and any point in the pipeline is $\vec{r}$; the angle with the x-axis is φ, the radial electric field ${\vec{E}}_{r}$, circumferential electric field ${\vec{E}}_{\varphi }$, axial electric field ${\vec{E}}_{z}$, radial magnetic field ${\vec{H}}_{r}$, circumferential magnetic field ${\vec{H}}_{\varphi }$, and axial magnetic field ${\vec{H}}_{z}$ of the TEmn mode in the pipeline are as follows:

(1) $${\vec{E}}_{r}=\pm j\frac{\omega \mu m}{k_{c}^{2}\vec{r}}H_{0}J_{m}\left(\frac{u_{mn}}{R}\vec{r}\right)\left(\begin{array}{l} \sin m\varphi \\ \cos m\varphi \end{array}\right)e^{-j\beta z}$$

(2) $${\vec{E}}_{\varphi }=j\frac{\omega \mu }{k_{c}}H_{0}J_{m}^{\prime }\left(\frac{u_{mn}}{R}\vec{r}\right)\left(\begin{array}{l} \cos m\varphi \\ \sin m\varphi \end{array}\right)e^{-j\beta z}$$

(3) $${\vec{E}}_{z}=0$$

(4) $${\vec{H}}_{r}=-j\frac{\beta }{k_{c}}H_{0}J_{m}^{\prime }\left(\frac{u_{mn}}{R}\vec{r}\right)\left(\begin{array}{l} \cos m\varphi \\ \sin m\varphi \end{array}\right)e^{-j\beta z}$$

(5) $${\vec{H}}_{\varphi }=\pm H_{0}\frac{j\beta m}{k_{c}^{2}\vec{r}}J_{m}\left(\frac{u_{mn}}{R}\vec{r}\right)\left(\begin{array}{l} \sin m\varphi \\ \cos m\varphi \end{array}\right)e^{-j\beta z}$$

(6) $${\vec{H}}_{z}=H_{0}J_{m}\left(\frac{u_{mn}}{R}\vec{r}\right)\left(\begin{array}{l} \cos m\varphi \\ \sin m\varphi \end{array}\right)e^{-j\beta z}$$

where *m* represents the wavenumber of the microwave propagation distributed along the circumference, and *n* represents the number of fields distributed along the radius, where ω is the angular frequency, μ is the permeability, $k_{c}$ is the cutoff wavenumber, and β is the propagation constant. $H_{0}$ is the peak value of the magnetic field during propagation of the TEmn mode. It can be observed from the above formula that the TEmn wave in the pipeline propagates sinusoidally along the axial direction. $J_{m}$ is the m-order Bessel function of the first kind; $J_{m}^{\prime }$ is the derivative of the m-order Bessel function of the first kind; and umn is the root of the TE01-modulus Bessel function.

When *m* = 0, and *n* = 1, the microwave wave number distributed along the circumference of the TE01 mode is 0, and the microwave wave number distributed along the radius is 1. Therefore, the TE01 mode has circular symmetry in the circumferential direction, the loss in the circumferential direction is the smallest, and loss is the smallest near the pipe wall, which is suitable for long-distance transmission. The use of TE01 mode microwave to detect small-diameter pipes can realize long-distance, small-diameter pipe detection.

The pipe wall current is related to the tangential magnetic field in the pipe wall. Because the TE01 mode has only the Hz component near the pipe wall, the pipe wall current of the TE01 mode is:

(7) $${\vec{J}}_{s}=\vec{n}\times {\vec{H}}_{z}=-\vec{r}\times {\vec{H}}_{z}=H_{z}{\vec{e}}_{\varphi }$$

where $\vec{n}$ is the unit normal vector of the inner surface of the metal waveguide; and ${\vec{H}}_{t}$ is the tangential magnetic field around the inner wall. The pipe wall current propagates along the pipe wall. When there is a defect in the pipe, the defect hinders the pipe wall current propagation, and return loss occurs during the propagation of the microwave in the pipe so that a pipe wall defect can be detected.

### 2.2. Microwave Detection Mechanism of TE01 Mode for Pipeline Defects

From Equations (1)–(6), when the TE01 mode propagates in the pipeline, there are inner wall defects in the pipeline, and defect depth *h*, defect width *l*, and the field distribution equation of the TE01 mode microwave at the defect are:

(8) $${\vec{E}}_{r}=\pm j\frac{\omega \mu m}{k_{1c}^{2}\vec{r}}H_{10}J_{m}\left(\frac{\mu_{mn}}{R+h}\vec{r}\right)\left(\begin{array}{l} \sin m\varphi \\ \cos m\varphi \end{array}\right)e^{-j\beta l}$$

(9) $${\vec{E}}_{\varphi }=j\frac{\omega \mu }{k_{1c}}H_{10}J_{m}^{\prime }\left(\frac{\mu_{mn}}{R+h}\vec{r}\right)\left(\begin{array}{l} \cos m\varphi \\ \sin m\varphi \end{array}\right)e^{-j\beta l}$$

(10) $${\vec{E}}_{z}=0$$

(11) $${\vec{H}}_{r}=-j\frac{\beta }{k_{1c}}H_{10}J_{m}^{\prime }\left(\frac{\mu_{mn}}{R+h}\vec{r}\right)\left(\begin{array}{l} \cos m\varphi \\ \sin m\varphi \end{array}\right)e^{-j\beta l}$$

(12) $${\vec{H}}_{\varphi }=\pm H_{10}\frac{j\beta m}{k_{1c}^{2}\vec{r}}J_{m}\left(\frac{\mu_{mn}}{R+h}\vec{r}\right)\left(\begin{array}{l} \sin m\varphi \\ \cos m\varphi \end{array}\right)e^{-j\beta l}$$

(13) $${\vec{H}}_{z}=H_{10}J_{m}\left(\frac{\mu_{mn}}{R+h}\vec{r}\right)\left(\begin{array}{l} \cos m\varphi \\ \sin m\varphi \end{array}\right)e^{-j\beta l}$$

Equations (8) to (13) show that when a defect occurs in the pipeline, the field equation propagating at the defect changes. When there is a wall defect with a depth of h in the pipeline, the radius of the pipeline at the defect increases, the electric field and magnetic field components transmitted in the pipeline decrease, and the microwave produces energy loss at the defect in the pipeline.

### 2.3. Microwave Mode Distortion at Defects

When there is no defect in the pipeline, the TE01 mode microwave is stably transmitted in the pipeline, and no mode jump occurs. When a defect occurs on the inner wall of the pipeline, the diameter of the defect changes, and a new microwave propagation mode jump occurs at the defect. The defect depth is 5 mm as an example to verify the microwave detection capability of TE01 mode. At this time, the radius of the defect is 33 mm, and the propagation mode of microwave is TM12 mode. When the TE01 mode is coupled to the TM12 mode, the coupling coefficient is:

(14) $$C=\frac{2mJ_{m}\left(\mu_{mn2}\frac{R+h}{R}\right)}{\mu_{mn2}J_{m+1}\left(\mu_{mn2}\right)\sqrt{\left(v_{mn1}^{2}-m^{2}\right)}}$$

Therefore, the coupling coefficient propagating in the pipeline is related to the radius of the pipeline. The radius of the pipeline defect increases, and the microwave propagation mode is distorted to the higher-order mode. With the increase of the width of the pipeline defect, the propagation distance of the distortion field in the pipeline increases. Mode coupling with different strengths and weaknesses occurs in the pipeline, resulting in changes in the distribution of microwave energy propagating in the pipeline.

### 2.4. Analysis of Feature Quantity for TE01 Mode Microwave Defect Detection

When a microwave is transmitted in the pipeline, after passing through the short-circuit piece, a reflected wave is formed in the pipeline. The ratio of the microwave reflected wave to the incident wave is the reflection coefficient.

(15) $$\Gamma =\frac{{\vec{E}}^{-}}{{\vec{E}}^{+}}$$

It can be seen from Equations (1)–(3) that when the TE01 mode microwave propagates in the pipeline, the electric field propagates only in the φ direction in the pipeline; when the defect causes the microwave to propagate in the pipeline, the defect causes the propagation radius of the microwave to change when it propagates in the pipeline, and the propagation of the microwave in the pipeline is distorted, from Formula (1)–(3) and Formula (8)–(10); when the distortion mode at the defect is TE mode, it can be known that the reflection coefficient of microwave at the defect is:

(16) $$\Gamma =\frac{k_{c}\left[\begin{array}{cc} 0 & -\mu_{1}\\ \mu_{1} & 0 \end{array}\right]\left[\begin{array}{c} H_{10}k_{1c}J_{m}^{\prime }\left(\frac{u_{mn}}{R+h}\right)\left(\begin{array}{c} \cos m\varphi \\ \sin m\varphi \end{array}\right)\left(e^{-j\beta l}\right)\\ \pm \frac{1}{r}\mu_{1}H_{10}J_{m}\left(\frac{u_{mn}}{R+h}\right)\left(\begin{array}{c} \sin m\varphi \\ \cos m\varphi \end{array}\right)\left(e^{-j\beta l}\right) \end{array}\right]}{k_{1c}^{2}\mu H_{0}J_{m}\left(\frac{u_{mn}}{R}\right)\left(\begin{array}{c} \cos m\varphi \\ \sin m\varphi \end{array}\right)e^{-j\beta z}}$$

As shown in Equation (16), when the distortion wave at the defect is a TE wave, the reflection coefficient is the ratio of the electric field of the reflected wave to the electric field of the incident wave. At this time, when a defect occurs in the pipe, the propagation distance of the microwave in the pipe and the pipe diameter changes, resulting in a change in the reflection coefficient.

When the distortion mode at the defect in the pipeline is the TM mode, the reflection coefficient of the microwave is:

(17) $$\Gamma =\frac{\left[\begin{array}{ccc} -\frac{j\beta }{k_{c}} & 0 & 0\\ 0 & \pm \frac{j\beta m}{k_{c}^{2}} & 0\\ 0 & 0 & 1 \end{array}\right]\left[\begin{array}{c} E_{0}J_{m}^{\prime }\left(\frac{v_{mn}}{R+h}\vec{r}\right)\left(\begin{array}{c} \cos m\varphi \\ \sin m\varphi \end{array}\right)e^{-j\beta l}\\ E_{0}J_{m}\left(\frac{v_{mn}}{R+h}\vec{r}\right)\left(\begin{array}{c} \sin m\varphi \\ \cos m\varphi \end{array}\right)e^{-j\beta l}\\ E_{0}J_{m}\left(\frac{v_{mn}}{R+h}\vec{r}\right)\left(\begin{array}{c} \cos m\varphi \\ \sin m\varphi \end{array}\right)e^{-j\beta l} \end{array}\right]}{j\frac{\omega \mu }{k_{c}}H_{0}J_{m}^{\prime }\left(\frac{u_{mn}}{R}\vec{r}\right)\left(\begin{array}{l} \cos m\varphi \\ \sin m\varphi \end{array}\right)e^{-j\beta z}}$$

It can be seen from Equations (16) and (17) that when there are no defects in the pipeline, only the TE01 mode exists in the pipeline, the incident and reflected waves are equal, and the reflection coefficient is equal to 1. When there is a defect in the pipeline, the propagation mode in the pipeline is distorted, and the reflection coefficient is less than 1. Where l is the propagation distance of the microwave in the pipe, when there is a defect in the pipe, h is the depth of the defect, and the microwave reflection coefficient is related to the length and width of the defect. When there is a defect in the pipeline, the radius of the pipe wall at the defect changes, causing the electric field and magnetic field equations at the defect to change, and the electric field and magnetic field values of the reflected wave decrease. The microwave reflection coefficient was less than 1.

When there is a defect in a pipeline, the energy of the microwave source is absorbed at the defect, resulting in energy loss. The energy change in the pipeline is represented by the return loss.

(18) RL=−20lg|Γ|dB

When the inner wall of the pipeline is smooth and defect-free, the microwave is totally reflected in the pipeline without energy loss, and the return loss value is 0. When the inner wall of the pipeline is defective, part of the energy in the microwave transmission process is absorbed by the defect, resulting in energy loss and echo in the pipeline. The absolute value of the loss is greater than zero, and the return loss of the microwave reflected wave reflects the energy loss of the microwave when it is transmitted in the pipeline.

## 3. Microwave Propagation Laws at Defects on Inner Wall of Small-Diameter Pipes

**Figure 3:**
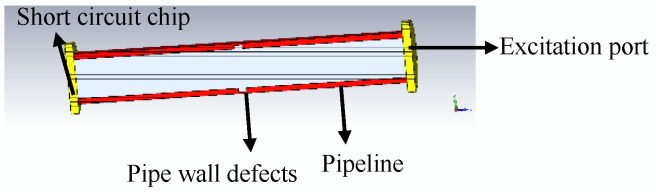
Establishment of simulation model.

The microwave detection model of TE01 mode small-diameter pipeline is established to test the ability of microwave to detect defects on the inner wall of the pipeline. The distribution of electric field, magnetic field, and pipe wall current in the pipeline is analyzed, and the influence of defects on the electric field and magnetic field current propagation in the pipe wall is analyzed. The simulation model is shown in Figure 3.

The energy distribution of the electric field, magnetic field, and pipe wall current in the pipeline is calculated by the finite integration method, and the microwave detection simulation model of the TE01 mode microwave small-diameter pipeline is established. The defect is 5 mm deep, 0 mm to 8 mm wide, the boundary conditions are set to ideal electrical boundary, the excitation method is single-port excitation, the pipe terminal is equipped with a short circuit, the reflection-terminal short-circuit method is used to detect small-diameter pipes, and the excitation frequency is 7.2–8.6 GHz; using the TE01 mode, the microwave is propagated in the pipeline, and the defect is detected by the microwave reflection method.

### 3.1. Analysis of Influence Law of Defects in Pipeline on Microwave Propagation

**Figure 4:**
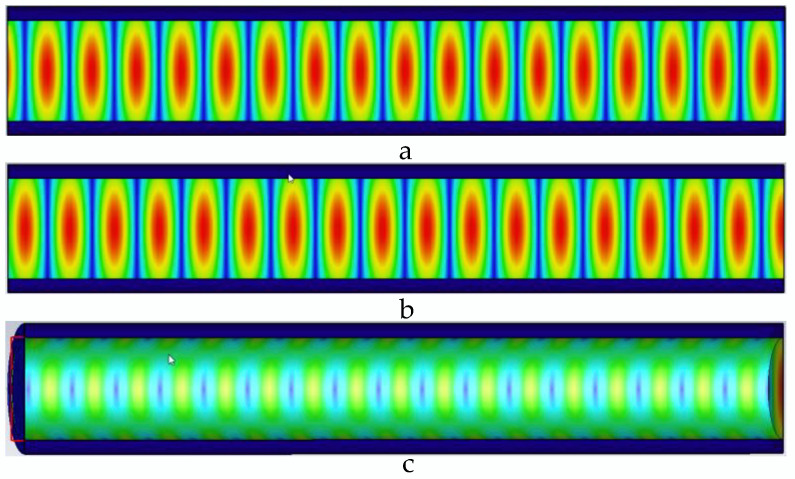
Distribution of electric field, magnetic field, and pipe wall current in the pipeline without defects. (**a**) Electric field distribution in the pipeline without defects; (**b**) magnetic field distribution in the pipeline without defects; and (**c**) current distribution of the pipe wall in the pipeline without defects.

The microwave in the pipeline propagates along the axial direction of the pipeline. When there is no defect in the pipeline, the microwave propagates periodically in the pipeline; when there is a defect in the pipeline, the defect destroys the microwave that propagates periodically in the pipeline, and the energy is lost at the defect, and defects in pipes can be detected. Distribution of electric field, magnetic field, and pipe wall current in the pipeline without defects is shown in Figure 4.

When there is no defect in the pipe, as shown in Figure 4, the microwave field distribution periodically propagates along the axis of the pipe, the wall current propagates along the pipe wall, and the propagation period is consistent with the magnetic field. In the pipe, the propagation period in is uniformly distributed. When the TE01 mode microwave is transmitted in the pipeline, it has circular symmetry, and the loss of the TE01 mode is small when it is transmitted in the pipeline. 

**Figure 5:**
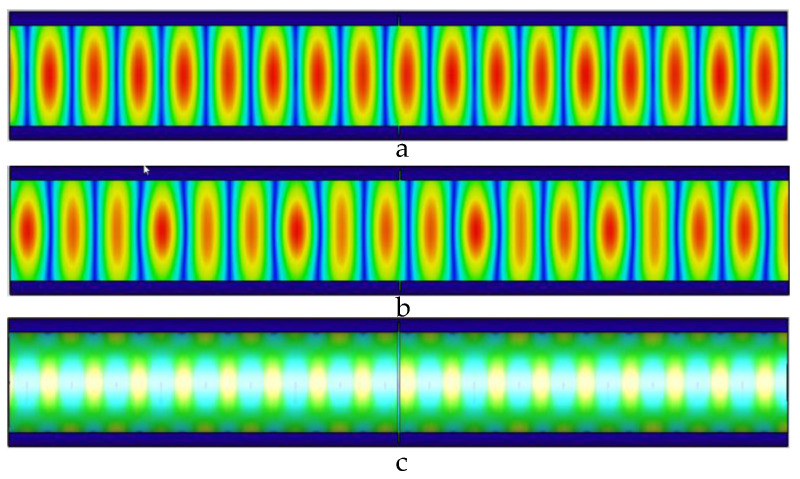
Distribution of electric field, magnetic field, and pipe wall current in the pipeline at 1 mm wide defect. (**a**) The electric field distribution in the pipeline with a 1 mm wide defect; (**b**) the magnetic field distribution in the pipeline with a 1 mm wide defect; and (**c**) the current distribution in the pipe wall with a 1 mm wide defect.

As shown in Figure 5, the defect width is 1 mm, the electric field causes a weak energy distribution change caused by the defect, and the magnetic field periodic distribution changes significantly; the leakage of energy at the defect causes uneven energy distribution in the pipeline, loss occurs at the defect, and microwaves are generated from the defect signal.

**Figure 6:**
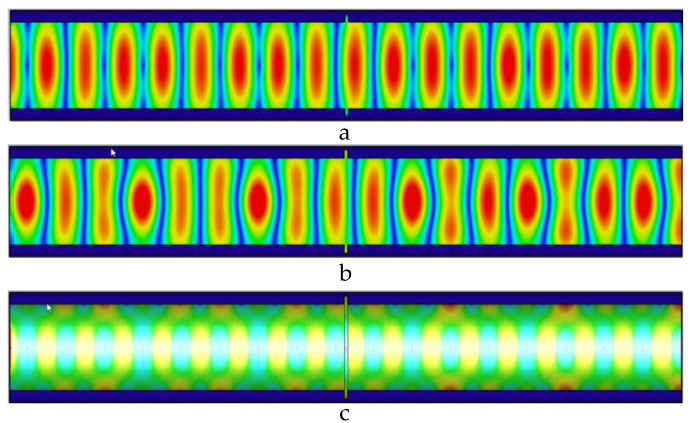
Distribution of electric field, magnetic field, and pipe wall current in the pipeline at 2 mm wide defect. (**a**) The electric field distribution in the pipeline with a 2 mm wide defect; (**b**) the magnetic field distribution in the pipeline with a 2 mm wide defect; and (**c**) the current distribution in the pipe wall with a 2 mm wide defect.

As shown in Figure 6, the width of the defect is 2 mm, the microwave propagation at the defect is destroyed, and the microwave forms a new propagation cycle in the pipeline. Defects lead to increased electric field, magnetic field, and wall current values propagating in the pipe.

**Figure 7:**
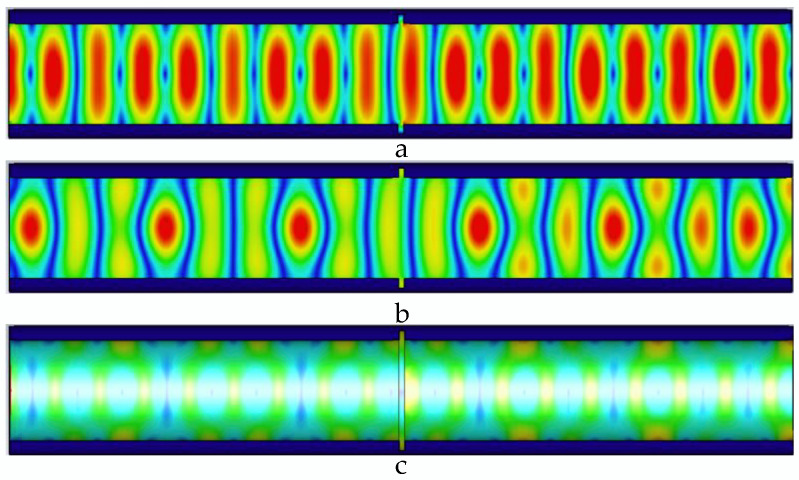
Distribution of electric field, magnetic field, and pipe wall current in the pipeline at 3 mm wide defect. (**a**) The electric field distribution in the pipeline with a 3 mm wide defect; (**b**) the magnetic field distribution in the pipeline with a 3 mm wide defect; and (**c**) the current distribution in the pipe wall with a 3 mm wide defect.

As shown in Figure 7, the width of the defect is 3 mm, and the defect causes the energy distribution in the pipeline to be more concentrated; and the propagation period and single-cycle energy distribution of the electric field, the magnetic field, and the wall current in the pipeline are obviously distorted; and the single-cycle energy distribution at the magnetic field and the wall current is obviously distorted. The propagation distance and energy distribution have changed significantly.

**Figure 8:**
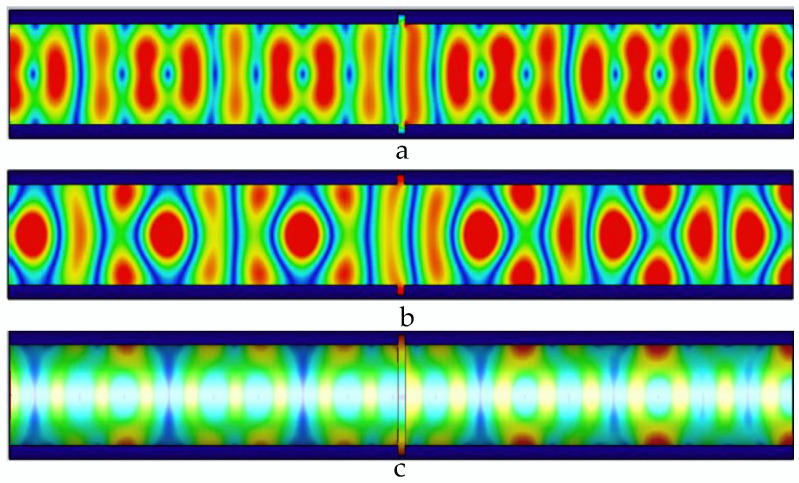
Distribution of electric field, magnetic field, and pipe wall current in the pipeline at 4 mm wide defect. (**a**) The electric field distribution in the pipeline with a 4 mm wide defect; (**b**) the magnetic field distribution in the pipeline with a 4 mm wide defect; and (**c**) the current distribution in the pipe wall with a 4 mm wide defect.

As shown in Figure 8, the defect width is 4 mm, and the microwave at the defect cannot form a complete cycle. The defect leads to an increase in the peak value of microwave energy propagating in the pipeline and a decrease in the propagation period.

**Figure 9:**
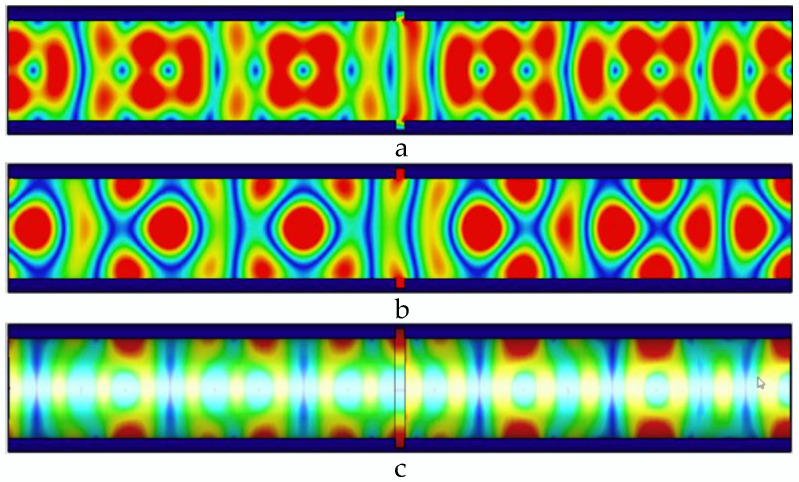
Distribution of electric field, magnetic field, and pipe wall current in the pipeline at 5 mm wide defect. (**a**) The electric field distribution in the pipeline with a 5 mm wide defect; (**b**) the magnetic field distribution in the pipeline with a 5 mm wide defect; and (**c**) the current distribution in the pipe wall with a 5 mm wide defect.

As shown in Figure 9, the defect width is 5 mm, the number of microwave propagation cycles in the pipeline decreases, the single-cycle microwave energy distribution in the pipeline is uneven, a complete propagation cycle cannot be formed at the defect, and the energy is concentrated, resulting in a large energy loss. 

**Figure 10:**
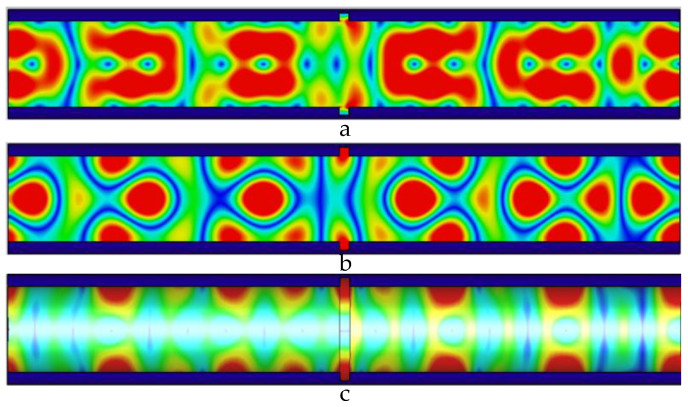
Distribution of electric field, magnetic field, and pipe wall current in the pipeline at 6 mm wide defect. (**a**) The electric field distribution in the pipeline with a 6 mm wide defect; (**b**) the magnetic field distribution in the pipeline with a 6 mm wide defect; and (**c**) the current distribution in the pipe wall with a 6 mm wide defect.

As shown in Figure 10, the defect width is 6 mm, and the defect causes the complete microwave propagation cycle in the pipeline to be reduced to 4. The energy during single-cycle propagation is more concentrated, and the defect causes the return loss value of the microwave reflected wave to increase.

**Figure 11:**
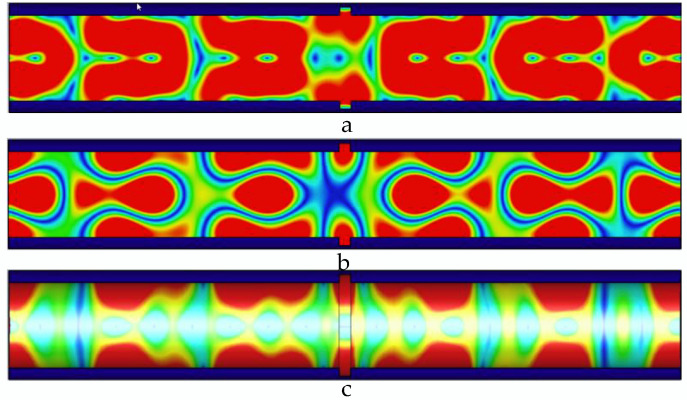
Distribution of electric field, magnetic field, and pipe wall current in the pipeline at 7 mm wide defect. (**a**) The electric field distribution in the pipeline with a 7 mm wide defect; (**b**) the magnetic field distribution in the pipeline with a 7 mm wide defect; the (**c**) the current distribution in the pipe wall with a 7 mm wide defect.

As shown in Figure 11, the defect width is 7 mm, a large amount of energy is accumulated at the defect, and a large energy loss is caused at the defect.

**Figure 12:**
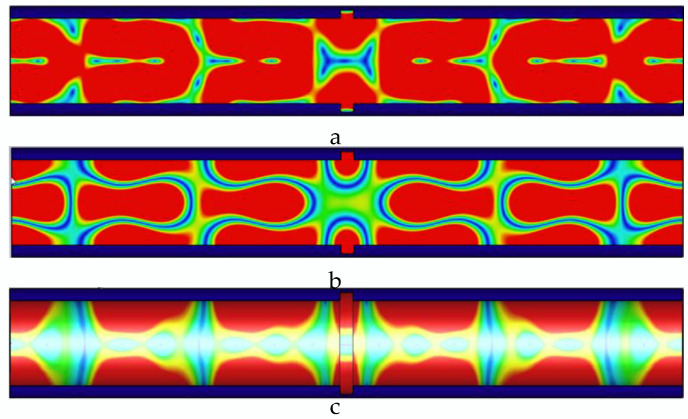
Distribution of electric field, magnetic field, and pipe wall current in the pipeline at 8 mm wide defect. (**a**) The electric field distribution in the pipeline with an 8 mm wide defect; (**b**) the magnetic field distribution in the pipeline with an 8 mm wide defect; and (**c**) the current distribution in the pipe wall with an 8 mm wide defect.

As shown in Figure 12, the defect width is 8 mm, the energy in the pipeline is concentrated at the pipeline defect, the number of microwave propagation cycles in the pipeline is reduced, and the single-cycle microwave energy distribution is more concentrated. The peak value of the field distribution energy in the pipeline increases, and the energy loss in the defect increases.

It can be seen from the field distribution in the pipeline that with the increase of the defect width, the uniformly propagated microwave field distribution in the pipeline is destroyed, the propagation period in the pipeline gradually decreases, the amplitude of the single-period field distribution gradually increases, and the energy at the defect accumulates more. As the defect width increases, when there is no defect in the pipeline, the TE01 mode has no microwave in the circumferential direction. There is a single microwave field distribution along the radial direction; with the increase of the defect width in the pipeline, the microwave produces energy loss at the defect and cannot travel a complete propagation cycle, a standing wave gradually appears in the circumferential direction, the microwave distribution along the radial direction fields is greater than 1, and defects cause microwave propagation mode jumps in the pipe. The electric field, magnetic field, and the peak value of the pipe wall current in the pipeline are shown in Table 1.

With the increase of defect width, the number of microwave propagation electric field cycles in the pipeline decreases, the peak value of single-cycle microwave increases, and the peak value of the propagating electric field in the pipeline increases. With the increase of the defect width in the pipeline, the peak value of the single-cycle peak of the magnetic field in the pipeline increases with the increase of the defect width, and the increase rate of the single-cycle peak of the magnetic field increases. When there is no defect in the pipeline, the peak value of the wall current of the TE01 mold is consistent with the peak value of the magnetic field in the pipeline; when there is a defect in the pipeline, a new boundary condition is established at the defect, and the wall current produces energy loss at the defect. The peak value of the pipe wall current is smaller than the peak value of the magnetic field in the pipe. As the defect width increases, the peak value of the wall current increases with the increase of the defect width. At this time, the inner wall defect is perpendicular to the current direction of the tube wall, resulting in the largest energy loss and the best defect detection effect.

### 3.2. Return Loss Analysis

**Figure 13:**
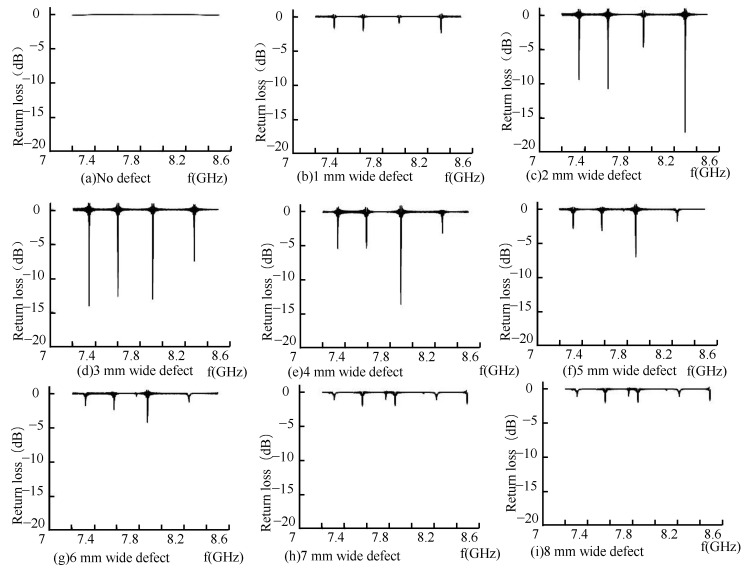
Reflection coefficient of reflected wave.

From the microwave energy field distribution in the pipeline, it can be known that the defects in the pipeline cause the distortion of the defect field distribution, which leads to the change of the period and energy distribution of the microwave propagating in the pipeline and the accumulation of defect energy, resulting in the return loss of the microwave reflected wave. The defect in the pipeline is the crack on the inner wall of the pipeline, the return loss of the microwave reflected wave is shown in the Figure 13.

**Figure 14:**
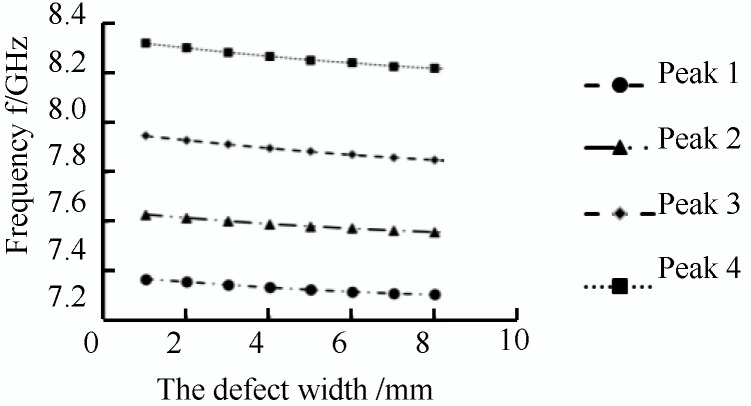
Shift of detection frequency at different width defects.

As shown in Figure 13a, when there are no defects in the pipeline, the inner wall of the pipeline is smooth, and the microwave forms a total reflection through the short-circuit plate in the pipeline. There was no energy loss in the pipeline; the return loss was zero, and the reflection coefficient was one. At this time, the incident wave electric field was equal to the reflected wave electric field. When there is a defect in the pipeline, the energy in the pipeline is attenuated, and the return loss occurs at the defect, as shown in Figure 13b–i, and the microwave reflected wave is detected in the TE01 mode as the width of the defect in the pipeline increases. There are multiple echo signals; the detection frequency of the echo signal is shifted in frequency, and the detection frequency shift at defects of different widths is shown in the Figure 14.

As shown in Figure 14, in the process of TE01 mode microwave small-diameter pipeline inspection, the microwave reflected wave has four peaks of the microwave echo signal with the appearance of the defect. As the defect width increases, the microwave detection frequency at the peak of the microwave echo signal decreases linearly, and the defect width increases with a step size of 1 mm. In TE01 mode, setting the appropriate propagation frequency in the actual detection process can detect defects of different widths in the pipeline.

## 4. Experimental Analysis

**Figure 15:**
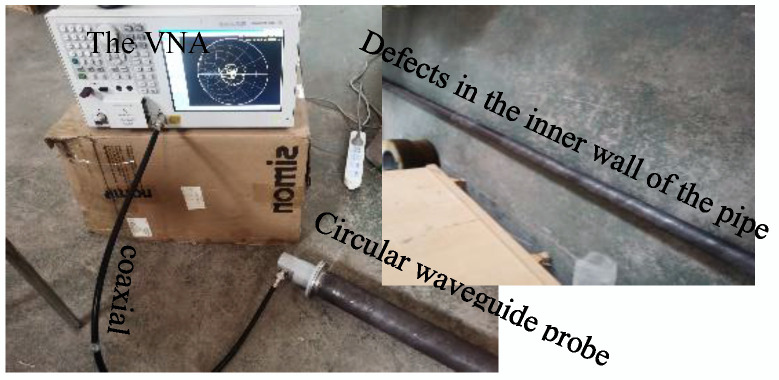
Experimental platform for small-diameter pipeline inspection.

An experimental platform for microwave detection of TE01 mold small-diameter pipes was developed. The experimental platform consisted of a vector network analyzer, coaxial cables, pipes with inner wall defects of different widths, and waveguide probes. The width of the defect on the inner wall of the pipeline was 1–8 mm, the depth of the defect was 5 mm, the diameter of pipe was 66 mm, and the length of the pipeline was 4 m. The microwave propagation frequency is 3.5 GHz–8.5 GHz in the experiment. The experimental platform for small- diameter pipeline inspection is shown in the Figure 15.

The vector network analyzer transmits and receives microwaves through a coaxial cable and transmits microwaves at one end of the pipeline as a microwave-transmitting port through a circular waveguide probe. The analyzer extracts the microwave return loss and feeds back the energy change in the microwaves in the pipeline.

**Figure 16:**
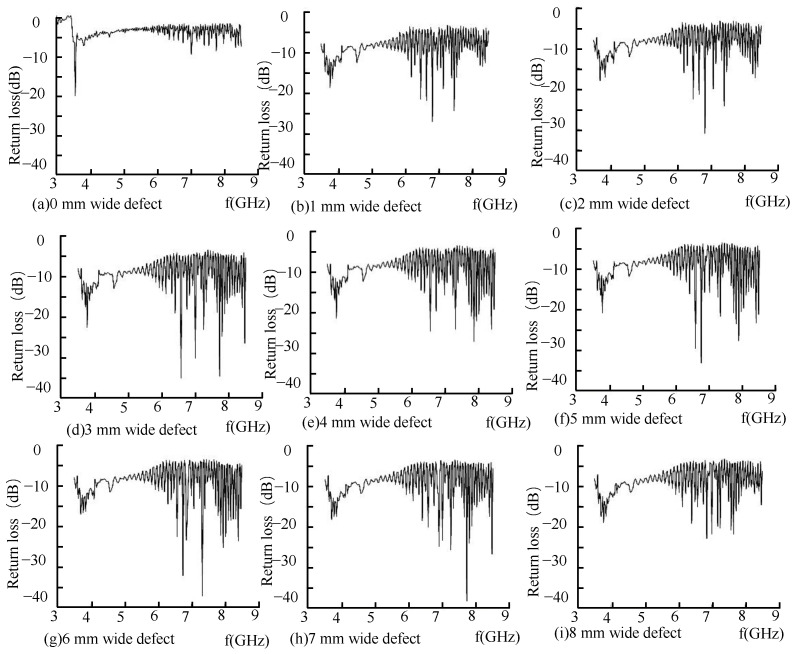
Reflection coefficient of reflected wave.

Under different defect widths, small-diameter pipes with defects of different widths are inspected, and the return loss of the reflected wave is shown in Figure 16.

**Figure 17:**
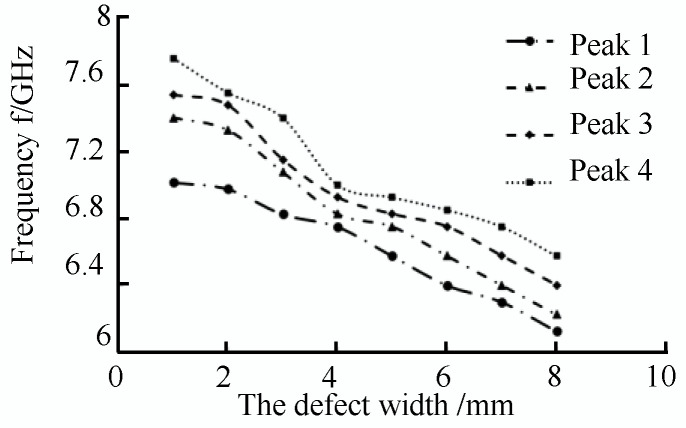
Shift of detection frequency at different width defects.

As shown in Figure 16a, when there is no defect in the pipe, the inner wall of the pipe is not smooth, the echo signal of the microwave reflected wave has energy loss, and the base value of the return loss is –5dB. In the case of ring-shaped defects, because the direction of the defect is perpendicular to the propagation direction of the current on the tube wall, the propagation of the current on the tube wall is hindered, and the energy loss in this direction is the largest. It can be observed from Figure 16b that when there is a defect in the pipeline, the fundamental value of the microwave reflected wave signal is reduced to 10dB. When there is no defect in the pipeline, the cut-off frequency of the pipeline is 3.55GHz. When there are 5 mm deep defects in the pipeline, the defects of different widths cause a jump in the propagation mode in the pipeline, the cut-off frequency is 3.85GHz.It can be seen from Figure 16c–i that with an increase in the defect width in the pipeline, the microwave detection frequency gradually decreases. The maximum peak point in the pipeline is extracted, and the relationship between the defect width and detection frequency is shown in Figure 17.

Figure 17 shows that in the TE01 propagation mode, with the increase of the defect width, the defect detection frequency decreases linearly; the TE01 mode microwave can detect the inner wall defects of small-diameter pipes and is most sensitive to the circumferential defects of the inner wall of the pipe. The experiment and simulation are in good agreement.

## 5. Conclusions

(1) The transmission loss of the TE01 mode microwave in the pipeline is small, and it is suitable for long-distance transmissions of more than 100 m. By establishing a microwave detection model for small-diameter pipeline defects in the TE01 mode, the relationship between the change in defect size and the characteristic parameters of microwaves is obtained, and the microwave reflection coefficient and return loss can be used as the basis for microwave detection of small-diameter pipes.
(2) As the width of the inner wall defect in the pipeline increases, the number of propagation cycles of the electric field, magnetic field, and wall current in the pipeline decreases; the peak value of the single-cycle field increases; and the energy accumulated at the defect results in loss, and the return loss absolute value increased.
(3) When there is a defect in the pipeline, the fundamental value of the return loss decreases as a whole. The fundamental value of the return loss of the microwave reflected wave decreases with the existence of the defect, and with an increase in the defect width, the detection frequency of the microwave reflected wave at the defect decreases linearly. The TE01 mode microwave has a good detection ability for defects in the inner wall of the pipeline.

In the actual small-diameter pipeline inspection process, whether there is a defect in the pipeline is determined by the change in the base value of the microwave reflected wave and the change in the cut-off frequency of the microwave. The width of the pipeline defect is analyzed by the movement of the peak value of the echo signal of the microwave reflected wave.

## Figures and Tables

**Table 1:** Peak value of TE01 microwave field distribution in pipe.

The width of defect mm	0	1	2	3	4	5	6	7	8
The value of electric field V/m	1291.91	1317.67	1461.68	1869.49	2368.71	2961.84	3885.45	4761.93	6168.96
The value of magnetic field A/m	3.00	3.25	3.62	4.22	5.13	6.36	8.54	11.14	15.00
The value of wall current A/m	3.00	2.81	2.86	3.24	3.94	5.03	6.79	9.25	12.63

## Data Availability

Not applicable.
